# A Comparative Analysis of Six Machine Learning Models Based on Ultrasound to Distinguish the Possibility of Central Cervical Lymph Node Metastasis in Patients With Papillary Thyroid Carcinoma

**DOI:** 10.3389/fonc.2021.656127

**Published:** 2021-06-25

**Authors:** Ying Zou, Yan Shi, Jihua Liu, Guanghe Cui, Zhi Yang, Meiling Liu, Fang Sun

**Affiliations:** ^1^ Department of Radiology, First Teaching Hospital of Tianjin University of Traditional Chinese Medicine, Tianjin, China; ^2^ Department of Ultrasonography, Binzhou Medical University Hospital, Binzhou City, China

**Keywords:** machine learning, papillary thyroid carcinoma, lymphatic metastasis, ultrasound, random forest

## Abstract

Current approaches to predict central cervical lymph node metastasis (CLNM) in patients with papillary thyroid carcinoma (PTC) have failed to identify patients who would benefit from preventive treatment. Machine learning has offered the opportunity to improve accuracy by comparing the different algorithms. We assessed which machine learning algorithm can best improve CLNM prediction. This retrospective study used routine ultrasound data of 1,364 PTC patients. Six machine learning algorithms were compared to predict the possibility of CLNM. Predictive accuracy was assessed by sensitivity, specificity, positive predictive value, negative predictive value, and the area under the curve (AUC). The patients were randomly split into the training (70%), validation (15%), and test (15%) data sets. Random forest (RF) led to the best diagnostic model in the test cohort (AUC 0.731 ± 0.036, 95% confidence interval: 0.664–0.791). The diagnostic performance of the RF algorithm was most dependent on the following five top-rank features: extrathyroidal extension (27.597), age (17.275), T stage (15.058), shape (13.474), and multifocality (12.929). In conclusion, this study demonstrated promise for integrating machine learning methods into clinical decision-making processes, though these would need to be tested prospectively.

## Introduction

According to the American Cancer Society 2020 American Cancer Data Statistics, thyroid cancer incidence has accounted as the fifth leading cause of cancer in women (1), similar to China (2). Cervical lymph node metastasis is considered as a risk factor for recurrence (3, 4), of which central cervical lymph node metastasis (CLNM) is the most common (5). According to the American Thyroid Association (ATA) management guidelines for adult patients with thyroid cancer (6), whether there is CLNM or not directly affects the formulation of preoperative surgical procedures. Prophylactic central lymph node dissection (CLND) for cN0 papillary thyroid carcinoma (PTC) patients will undoubtedly cause excessive medical treatment. Therefore, it is of great significance to distinguish CLNM with non-invasive methods before surgery for the treatment and prognosis in PTC patients.

Ultrasound remains the most critical imaging modality in the evaluation of thyroid cancer according to the ATA Statement on Preoperative Imaging for Thyroid Cancer Surgery (7) due to its convenience, non-invasive, and non-radiation. However, it is challenging to detect CLNM due to the interference of the gas in the trachea and esophagus, and its diagnostic sensitivity is only about 20–40% (8–13).

In recent years, artificial intelligence (AI) in medicine has grown significantly as a state-of-the-art data analysis tool (14). In particular, radiology lends itself to AI because of its large digital data sets (15). Machine learning, a significant subset of AI, provides a great supporting role to improve diagnostic and prognostic accuracy (16). Recently, some studies have focused on machine learning to evaluate thyroid nodules (16–18) and lymph node metastasis in patients with thyroid cancer (19), and the highest area under the curve (AUC) can reach 0.953. The machine learning classifiers used in these studies include neural networks, decision trees, random forest (RF), and deep learning. However, few studies focus on comparing the diagnostic performance of various machine learning classifiers in evaluating CLNM in PTC patients.

In the current study, based on published literature (20–23), we hypothesized that the machine learning models based on ultrasound could achieve higher performance in predicting CLNM in PTC patients. The study’s purpose was, first, to develop machine learning-based models using six different classifiers to distinguish CLNM from non-CLNM based on preoperative ultrasound images. Second, to validate and test the diagnostic performance of the six models. Third, to compare the performance of six classifiers.

## Materials and Methods

### Patients Population

The research ethics committee of Binzhou Medical University Hospital approved this retrospective study (No. LW-024), and the requirement for written informed consent was waived since the retrospective nature. All the included data were anonymized. All patients’ medical records and ultrasound images were stored in the picture archiving and communication systems (PACS) of Binzhou Medical University Hospital. The clinicians could access the data by PACS. Considering patients’ privacy, you can contact the corresponding author to obtain all patients’ original data if necessary.

**Figure 1:**
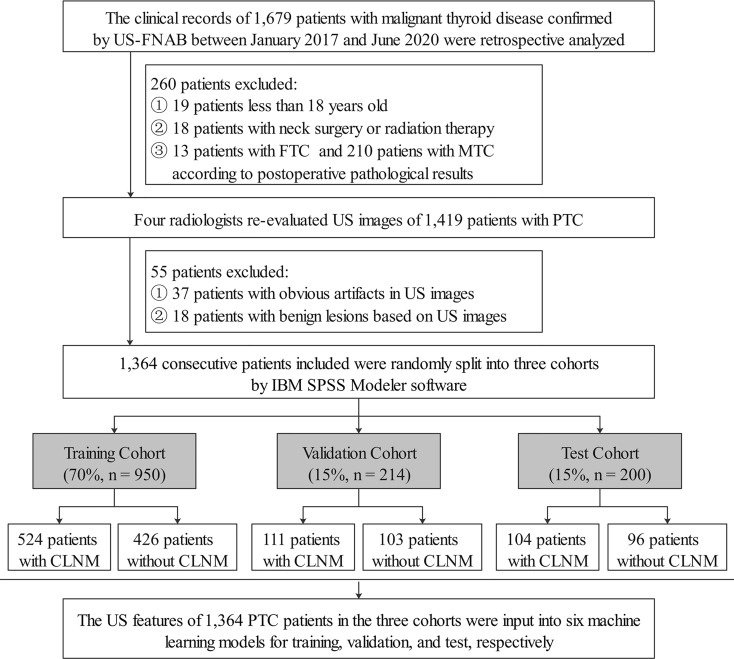
Flowchart showed inclusion and exclusion criteria of PTC patients in the current study. US-FNAB, ultrasound-guided fine-needle aspiration biopsy; FTC, follicular thyroid carcinoma; MTC, medullary thyroid carcinoma; US, ultrasound; PTC, papillary thyroid carcinoma; CLNM, central cervical lymph node metastasis.

The clinical records of 1,679 patients who visited Binzhou Medical University Hospital between January 2017 and June 2020 were retrospectively analyzed. The patients were treated for thyroid nodules and classified as Bethesda Categories V (suspicious for malignancy, risk of malignancy 50–75%) and VI (malignancy, risk of malignancy 97–99%) confirmed by ultrasound-guided fine-needle aspiration biopsy (US-FNAB). All patients underwent total thyroidectomy or thyroid lobectomy. CLND was performed for patients with preoperative ultrasound, suggesting the possible presence of CLNM; for cN0 patients, who were without evidence from preoperative imaging examination, CLND was conducted to follow the patients’ wishes after communication with the patients. Patients who did not undergo CLND were excluded from this study. Meanwhile, according to the ATA guidelines (24), for the lateral cervical lymph nodes, whether to perform lateral cervical lymph node dissection was based on preoperative imaging data. The inclusion criteria were as follows: patients with PTC confirmed by postoperative pathology and complete postoperative pathological results. We excluded patients with medullary thyroid carcinoma (MTC) and follicular thyroid carcinoma (FTC), less than 18 years old, previous thyroid operation or other neck surgery, and a history of radiation therapy. After a strict inclusion and exclusion process (**Figure 1**), a total of 1,364 consecutive patients were included, which were randomly split into the training (70%), validation (15%), and test (15%) data sets by IBM SPSS Modeler software (version 18.0). Demographics, including sex, age, final surgical pathology diagnosis, were thoroughly reviewed from the medical records.

### Image Acquisition and Analysis

All data were scanned using six different Doppler ultrasonic diagnostic apparatuses. The detailed ultrasound examination protocol was provided in the Appendix. Specific ultrasound diagnostic criteria of malignant thyroid lesions were according to the white paper of the American College of Radiology (ACR) Thyroid Imaging, Reporting, and Data System (TI-RADS) committee (25). Specific evaluation parameters included: location, background, T stage (diameter), margin, shape, composition, echogenicity, calcification, extrathyroidal extension (ETE), and multifocality. The ultrasound images were re-evaluated by four radiologists with 11, 12, 13, and 15 years of experience in thyroid cancer ultrasound diagnosis, blinded to clinical information and pathological diagnosis. A week later, the four radiologists took a second measurement and performed intra-observer consistency analysis. When the four radiologists disagreed with some image feature, it would be resolved through consultation with the fifth radiologist with more than 20 years of ultrasound diagnosis experience. Meanwhile, the inter-observer consistency analysis was performed. Consistency analysis was done by Cohen’s Kappa.

### Models Construction and Evaluation

By using the SPSS Modeler (version18.0, IBM, Armonk, New York), six classifiers, including decision tree (C5.0), logistic regression analysis (LRA), support vector machine (SVM), Bayesian network (BN), artificial neural network (ANN), and RF were used to establish the models. A brief description of these machine learning classifiers was shown in **Table A.1**. The parameters of classifiers played an essential role in the classification performance, and we set the parameters as follows to enhance the performance of each classifier: Boosting was used in the C5.0 algorithm to improve model accuracy; binomial logistic regression with backward selection was used in the LRA model; the radial basis function kernel was used as the kernel of the SVM, with the parameter C = 16, γ = 0.06 (1/number of features); BN adopted Markov chain structure and maximum likelihood parameter; a single-layered perceptron neural network for the ANN model consisted of one input layer, one or more hidden layers, and one output layer; 100 random tree number in RF model with the max feature included all the features input.

SPSS Modeler software randomly selected 70% of the data set as the training group and trained the model based on CLNM according to postoperative pathological results. 15% of the data set was applied as the validation group, and the remaining 15% was used as the test group to verify the trained model.

The data of the validation and test cohorts was input into the six machine learning models by SPSS Modeler to evaluate the diagnostic efficiency. Predictive performance was assessed using the receiver operating characteristic (ROC) curve, sensitivity, specificity, positive predictive value (PPV), and negative predictive value (NPV).

In addition, we further compared the concordance between the CLNM status as assessed by the best classifier in the six machine learning algorithms and the radiological CLNM status set at the time of the patient’s original treatment.

A power calculation was performed to ensure that both the validation and test data sets were sufficient to evaluate the AUC estimated from the training group.

### Statistical Analysis

All the statistical analysis was performed with SPSS (version 25.0, IBM, Armonk, New York), SPSS Modeler (version 18.0), and Medcalc Statistical Software (version 18.2.1). SPSS Modeler, GraphPad prism 8.3.0, and Medcalc Statistical Software were used to draw graphs. A *P*-value <0.05 was considered to be statistically significant. Use χ^2^ test to compare the differences in count data. Medcalc Statistical Software was used to calculate the six models’ AUCs and evaluate the predictions. The DeLong method was used to compare the AUCs of the six machine learning classifiers. Cohen’s kappa value was used to analyze the concordance between the best classifier and the radiologist’s assessment of CLNM. PASS 15 (Power Analysis and Sample Size Software, 2017, NCSS, LLC. Kaysville, Utah, USA, ncss.com/software/pass) “Tests for One ROC Curve” function was used to perform power calculation.

## Results

### Patient Demographics and Ultrasound Features

A total of 1,364 consecutive patients with PTC based on postoperative pathological results from January 1, 2017 to June 30, 2020 in our hospital were included in this retrospective analysis. We divided the patients into three parts randomly by IBM SPSS Modeler software: approximately 70% were conducted as the training cohort, about 15% were conducted as the validation cohort, and the remaining around 15% were used as the test cohort. Ultimately, the training cohort consisted of 950 patients (mean age, 47.01 years ± 11.54), including 227 males (mean age, 46.21 years ± 12.74) and 723 females (mean age, 47.26 years ± 11.13); and the validation cohort consisted of 214 patients (mean age, 47.08 years ± 11.09), including 48 males (mean age, 47.41 years ± 12.52) and 166 females (mean age, 46.00 years ± 10.61). A total of 200 consecutive PTC patients (mean age, 47.07 ± 10.63), including 51 males (mean age, 45.33 years ± 9.95) and 149 females (mean age, 47.57 years ± 10.79), were collected to form the test cohort.

Dummy variables were grouped according to the 8th American Joint Committee on Cancer (AJCC) staging systems (26) and ACR TI-RADS (25), details as follows: location (left lobe, right lobe, or isthmus), background (homogeneous or heterogeneous), diameter (T1a = “≤1 cm”, T1b = “1–2 cm”, T2 = “2–4 cm”, ≥T3 = “>4 cm”), margin (smooth, ill-defined, or lobulated/irregular), shape (wider-than-tall or taller-than-wide), composition (cystic, spongiform, mixed, or solid), echogenicity (anechoic, hyper/isoechoic, hypoechoic or very hypoechoic), calcification (none/large comet-tail, macrocalcification, rim calcification, or microcalcification), ETE, and multifocality. No statistically significant difference was observed among the three cohorts (*P >*0.05). Cohen’s Kappa values of intra- and inter-observe consistency analyses were all >0.8, indicating a solid consistency. Baseline epidemiologic and ultrasonic characteristics for the three cohorts were shown in **Table 1**.

**Table 1:** Comparison of clinical and ultrasonic characteristics of the PTC patients in the training, validation, and test cohorts.

	Training (n = 950)	Validation (n = 214)	Test (n = 200)	χ^2^	*P*
Sex				0.536	0.765
Male	227 (23.9%)	48 (22.4%)	51 (25.5%)		
Female	723 (76.1%)	166 (77.6%)	149 (74.5%)		
Age^*^				0.688	0.709
≤55	461 (48.5%)	105 (49.1%)	91 (45.5%)		
>55	489 (51.5%)	109 50.9(%)	109 (54.5%)		
Location				8.855	0.065
Left lobe	294 (30.9%)	76 (35.5%)	63 (31.5%)		
Right lobe	340 (35.8%)	83 (38.8%)	60 (30%)		
Isthmus	316 (33.3%)	55 (25.7%)	77 (38.5%)		
Background				2.940	0.230
Homogeneous	553 (58.2%)	111 (51.9%)	112 (56%)		
Heterogeneous	397 (41.8%)	103 (48.1%)	88 (44%)		
T stage^**^				17.796	0.065
T1a	523 (55.1%)	96 (44.9%)	121 (60.5%)		
T1b	301 (31.7%)	90 (42.1%)	51 (25.5%)		
T2	106 (11.2%)	26 (12.1%)	27 (13.5%)		
≥T3	20 (2.1%)	2 (0.9%)	1 (0.5%)		
Margin^***^				5.134	0.274
Smooth	23 (2.4%)	8 (3.7%)	3 (1.5%)		
Ill-defined	98 (10.3%)	30 (14.0%)	25 (12.5%)		
Lobulated/irregular	829 (87.3%)	176 (82.2%)	172 (86%)		
Shape^***^				4.739	0.094
Wider-than-tall	271 (28.5%)	75 (35.0%)	52 (26%)		
Taller-than-wide	679 (71.5%)	139 (65.0%)	148 (74%)		
Composition^***^					
Cystic	0	0	0	3.135	0.209
Spongiform	0	0	0		
Mixed	35 (3.7%)	5 (2.3%)	3 (1.5%)		
Solid	915 (96.3%)	209 (97.7%)	197 (98.5%)		
Echogenicity^***^				3.253	0.511
Anechoic	0	0	0		
Hyper/isoechoic	14 (1.5%)	6 (2.8%)	5 (2.5%)		
Hypoechoic	832 (87.6%)	184 (86.0%)	170 (85%)		
Very hypoechoic	104 (10.9%)	24 (11.2%)	25 (12.5%)		
Calcification^***^				4.309	0.651
None/large comet-tail	274 (28.8%)	58 (27.1%)	62 (31%)		
Macrocalcification	47 (4.9%)	8 (3.7%)	5 (2.5%)		
Rim calcification	4 (0.4%)	0	1 (0.5%)		
Microcalcification	625 (65.8%)	148 (69.2%)	132 (66%)		
Extrathyroidal extension				0.381	0.827
No	806 (84.8%)	183 (85.5%)	173 (86.5%)		
Yes	144 (15.2%)	31 (14.5%)	27 (13.5%)		
Multifocality				1.432	0.489
No	318 (33.5%)	64 (29.9%)	61 (30.5%)		
Yes	632 (66.5%)	150 (70.1%)	139 (69.5%)		

^*^We divided the patients into two groups based on age, using 55 years old as a cut-off value according to the 8th AJCC staging systems.

^**^T1a: ≤1 cm, T1b: 1–2 cm, T2: 2–4 cm, ≥ T3: >4 cm, according to the classification of diameter in the 8th AJCC staging systems.

^***^Refer to ACR TI-RADS.

PTC, papillary thyroid carcinoma; AJCC, American Joint Committee on Cancer; ACR, the American College of Radiology; TI-RADS, Thyroid Imaging, Reporting, and Data System.

### Diagnostic Performance of the Six Machine Learning Algorithms in the Training Cohort

**Figure 2:**
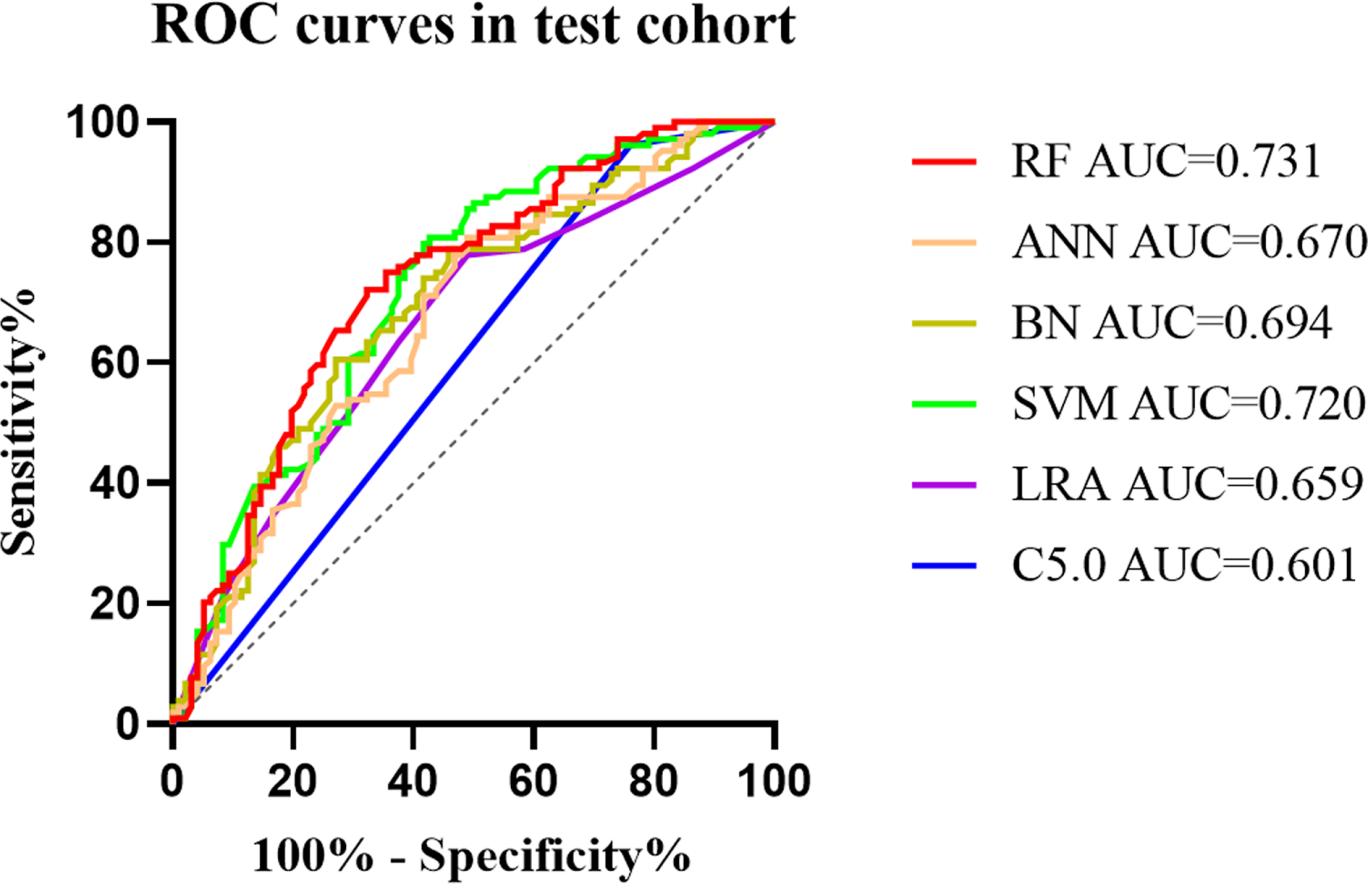
The mixed ROC curves of the six machine learning models in the test cohort. The RF algorithm had the highest AUC, indicating that it was the tremendous significance for the differentiation of CLNM and non-CLNM in patients with PTC. ROC, receiver operating characteristic; AUC, the area under the curve; C5.0, decision tree C5.0 algorithm; LRA, logistic regression analysis; SVM, support vector machine; BN, Bayesian network; ANN, artificial neural network; RF, random forest; CLNM, central cervical lymph node metastasis; PTC, papillary thyroid carcinoma.

**Figure 3:**
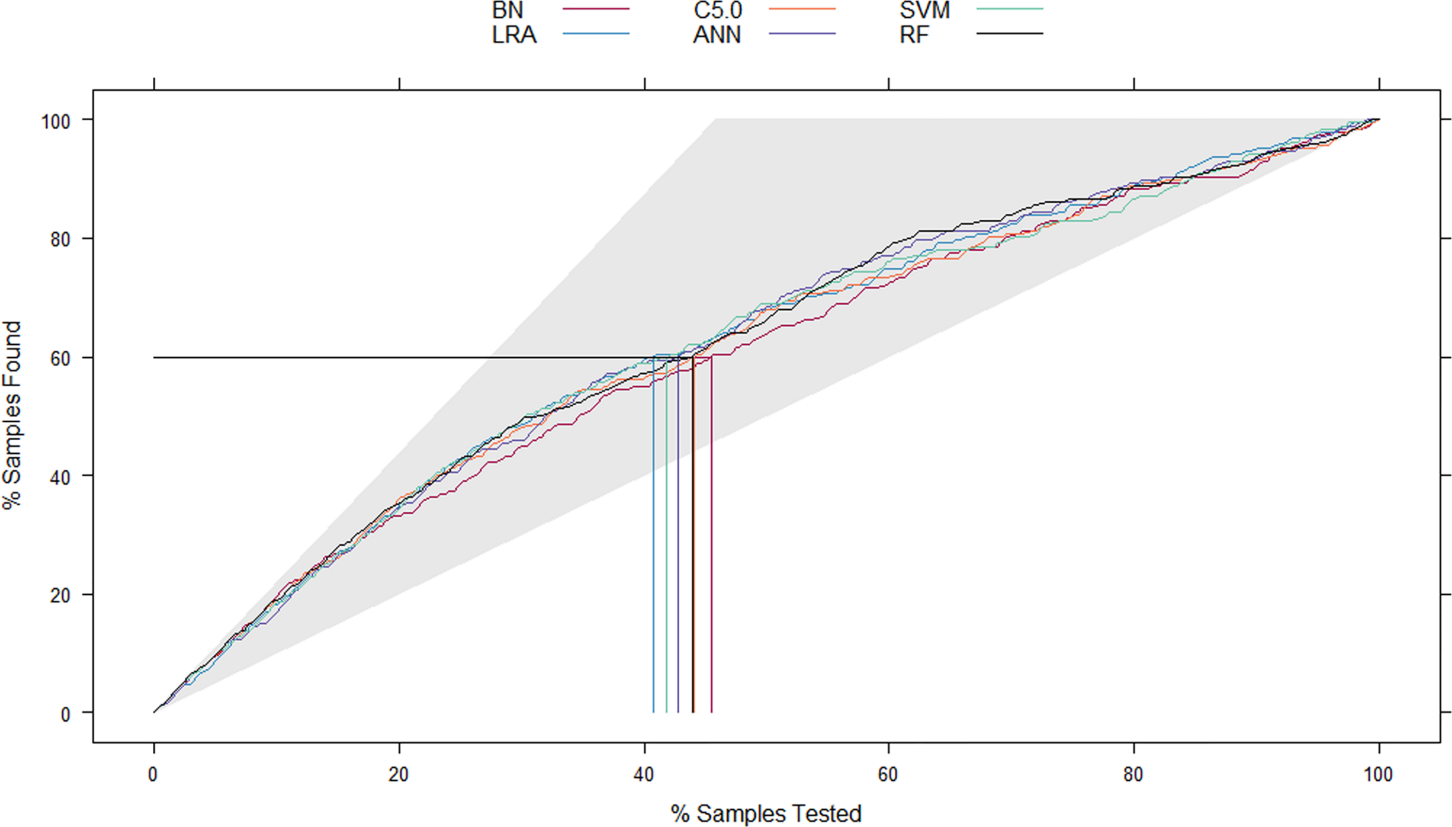
The mixed Lift curves of the six machine learning models in the test cohort. The Lift curve is one of the most commonly used methods for data mining classifiers. Unlike the ROC curve, Lift curve considers the classifier’s accuracy: the ratio of the number of positive classes obtains using the classifier to the number of positive classes obtains randomly without using the classifier. The Lift curve drawing process is similar to the ROC curve, and the difference is that Lift value and robust planar pose change in the opposite direction, which forms the opposite form of Lift curve and ROC curve. In the current mix Lift curves, RF also has the best diagnostic performance. ROC, receiver operating characteristic; RF, random forest.

The diagnostic performance of the six machine learning models was based on sex, age, and the ultrasound imaging features. In the training cohort, AUCs for C5.0, LRA, SVM, BN, ANN, and RF were 0.595 (95% confidence interval [CI]: 0.563–0.626), 0.732 (95% CI: 0.702–0.760), 0.776 (95% CI: 0.748–0.802), 0.748 (95% CI: 0.719–0.775), 0.731 (95% CI: 0.702–0.759), and 0.832 (95% CI: 0.807–0.855), respectively. Thus, the RF algorithm outperformed other machine learning algorithms in the training cohort (**Table 2** and **Figure A.1**). Application of the six machine-learning algorithms in the validation and test cohorts yielded high AUCs of RF algorithm with 0.754 (95% CI: 0.690–0.810) and 0.731 (95% CI: 0.664–0.791), which also proved the best diagnostic performance of RF (**Table 2**, **Figures 2**, **3**, and **A.2**). And the sensitivity, specificity, PPV, and NPV of the RF model in the validation cohort were 78.4% (95% CI: 69.6–85.6%), 68.0% (95% CI: 58.0–76.8%), 72.5% (95% CI: 66.2–78.0%), and 74.5% (95% CI: 66.6–81.0%), respectively. The sensitivity, specificity, PPV, and NPV of the RF model in the test cohort were 72.1% (95% CI: 62.5–80.5%), 67.7% (95% CI: 57.4–76.9%), 70.8% (95% CI: 63.9–76.8%), and 69.1% (95% CI: 61.5–75.9%), respectively (**Table 3**). The confusion matrices of the RF model in the training, validation, and test cohorts intuitively reflected the accuracy of the prediction (**Figure A.3**).

**Table 2:** Predictive performance of the six machine learning models for the training, validation, and test cohorts.

Model	Training cohort			Validation Cohort			Test Cohort		
	AUC ± SE	95% CI	*P*	AUC ± SE	95% CI	*P*	AUC ± SE	95% CI	*P*
C5.0	0.595 ± 0.012	0.563–0.626	<0.0001	0.585 ± 0.024	0.516–0.652	<0.0001	0.601 ± 0.024	0.529–0.669	0.0001
LRA	0.732 ± 0.016	0.702–0.760	<0.0001	0.701 ± 0.035	0.635–0.761	0.0350	0.659 ± 0.039	0.589–0.724	0.0083
SVM	0.776 ± 0.016	0.748–0.802	<0.0001	0.725 ± 0.035	0.660–0.784	0.1935	0.720 ± 0.036	0.653–0.781	0.5862
BN	0.748 ± 0.016	0.719–0.775	<0.0001	0.722 ± 0.035	0.657–0.781	0.2231	0.694 ± 0.037	0.625–0.757	0.0621
ANN	0.731 ± 0.016	0.702–0.759	<0.0001	0.713 ± 0.035	0.647–0.772	0.1588	0.670 ± 0.038	0.600–0.735	0.0054
RF	0.832 ± 0.013	0.807–0.855	<0.0001	0.754 ± 0.034	0.690–0.810	<0.0001	0.731 ± 0.036	0.664–0.791	0.0001

AUC, the area under the curve; SE, standard error; CI, confidence interval; C5.0, decision tree C5.0 algorithm; LRA, logistic regression analysis; SVM, support vector machine; BN, Bayesian network; ANN, artificial neural network; RF, random forest.

**Table 3:** Comparison the performance of the six machine learning models for the training, validation, and test cohorts.

Model	Sensitivity (%)	Specificity (%)	PPV (%)	NPV (%)
Training				
C5.0	93.3 (90.8–95.3)	25.6 (21.5–30.0)	60.7 (59.2–62.1)	75.7 (68.5–81.7)
LRA	76.9 (73.1–80.5)	59.4 (54.6–64.1)	70.0 (67.3–72.5)	67.6 (63.7–71.4)
SVM	89.3 (86.3–91.8)	52.8 (48.0–57.6)	70.0 (67.7–72.1)	80.1 (75.5–83.9)
BN	78.6 (74.9–82.1)	61.0 (56.2–65.7)	71.3 (68.6–73.8)	69.9 (66.0–73.6)
NN	82.4 (78.9–85.6)	54.0 (49.1–58.8)	68.8 (66.4–71.1)	71.4 (67.1–75.4)
RF	87.6 (84.5–90.3)	65.7 (61.0–70.2)	75.9 (73.3–78.3)	81.2 (77.3–84.5)
Validation				
C5.0	93.7 (87.4–97.4)	23.3 (15.5–32.7)	56.8 (53.9–59.7)	77.4 (60.7–88.4)
LRA	67.6 (58.0–76.1)	61.2 (51.1–70.6)	65.2 (58.8–71.2)	63.6 (56.2–70.5)
SVM	87.4 (79.7–92.9)	56.3 (46.2–66.1)	68.3 (63.1–73.1)	80.6 (71.2–87.4)
BN	88.3 (80.8–93.6)	47.6 (37.6–57.6)	64.5 (59.9–68.8)	79.0 (68.5–86.7)
NN	79.3 (70.5–86.4)	57.3 (47.2–67.0)	66.7 (61.1–71.8)	72.0 (63.2–79.3)
RF	78.4 (69.6–85.6)	68.0 (58.0–76.8)	72.5 (66.2–78.0)	74.5 (66.6–81.0)
Test				
C5.0	96.2 (90.4–98.9)	24.0 (15.8–33.7)	57.8 (54.9–60.7)	85.2 (67.4–94.1)
LRA	77.9 (68.7–85.4)	51.0 (40.6–61.4)	63.3 (57.8–68.4)	68.1 (58.6–76.3)
SVM	79.8 (70.8–87.0)	58.3 (47.8–68.3)	67.5 (61.6–72.8)	72.7 (63.7–80.2)
BN	60.6 (50.5–70.0)	72.9 (62.9–81.5)	70.8 (62.8–77.7)	63.1 (56.6–69.1)
NN	80.8 (71.9–87.8)	51.0 (40.6–61.4)	64.1 (58.8–69.1)	71.0 (61.2–79.2)
RF	72.1 (62.5–80.5)	67.7 (57.4–76.9)	70.8 (63.9–76.8)	69.1 (61.5–75.9)

Data in parentheses are 95% CI.

PPV, positive predictive value; NPV, negative predictive value; C5.0, decision tree C5.0 algorithm; LRA, logistic regression analysis; SVM, support vector machine; BN, Bayesian network; ANN, artificial neural network; RF, random forest; CI, confidence interval.

Cohen’s kappa value was 0.847, indicating strong concordance between the RF classifier and the radiologist’s assessment of CLNM.

### The Power Calculation

In the training set, the AUC of RF classifier was 0.832 (95% CI: 0.807–0.855), so we set the AUC0 = 0.83. In the validation and test sets, the AUCs were 0.754 (95% CI: 0.690–0.810) and 0.731 (95% CI: 0.664–0.791); so we set the AUC1 = 0.73-0.75, α = 0.05, False positive rate limited: 0.01–0.20. The result showed that when the target power was 0.80, the sample sizes of validation and test sets were 202 and 129, respectively. Therefore, both the validation and test sets were sufficient to evaluate the AUC estimated from the training set.

### The Relative Importance of Each Feature Within the RF Algorithm

**Figure 4:**
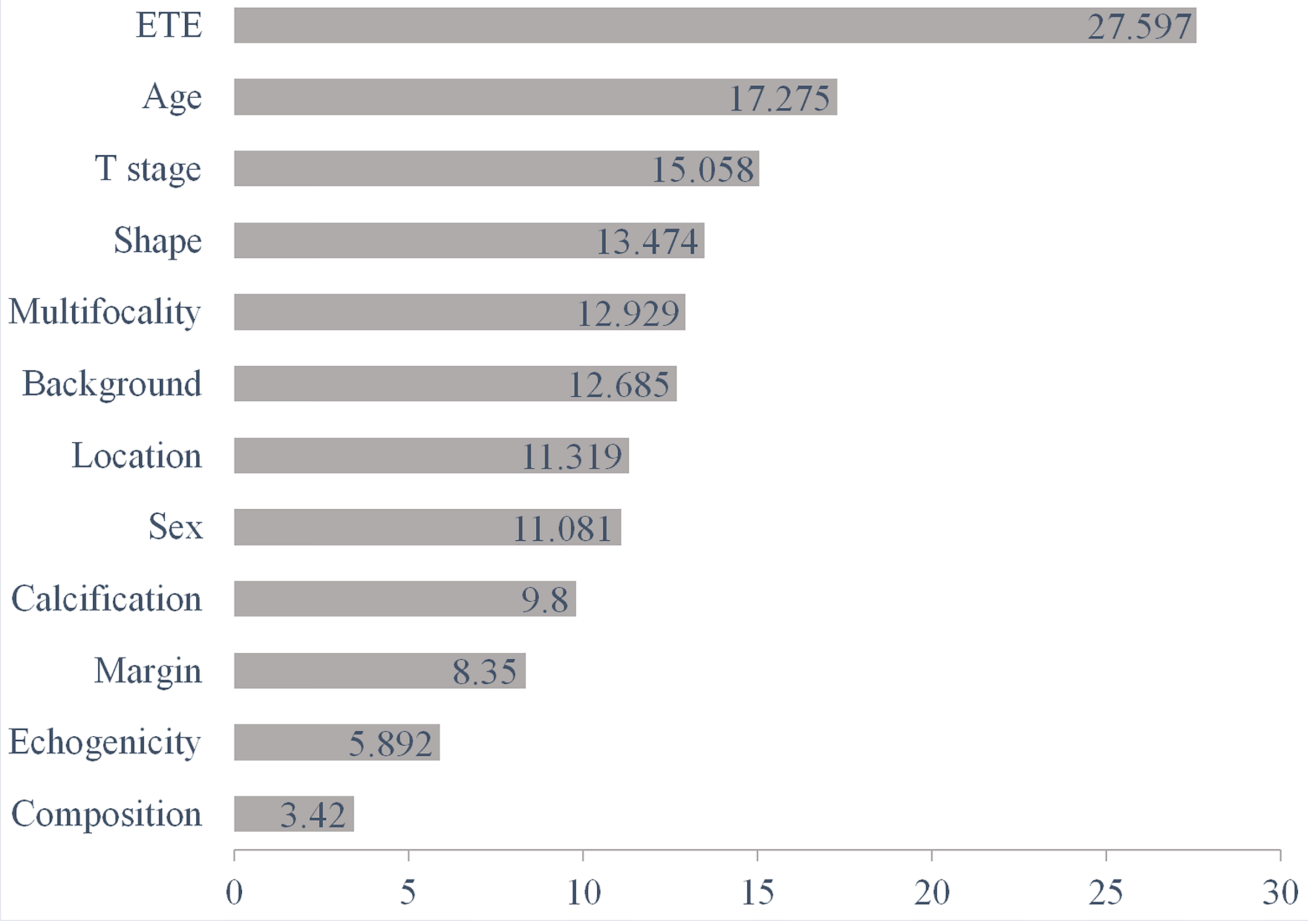
The diagnostic performance of the RF algorithm was most dependent on the following five top-rank features according to their mean decrease in Gini: ETE (27.597), age (17.275), T stage (15.058), shape (13.474), and multifocality (12.929). RF, random forest; ETE, extrathyroidal extension.

**Figure 5:**
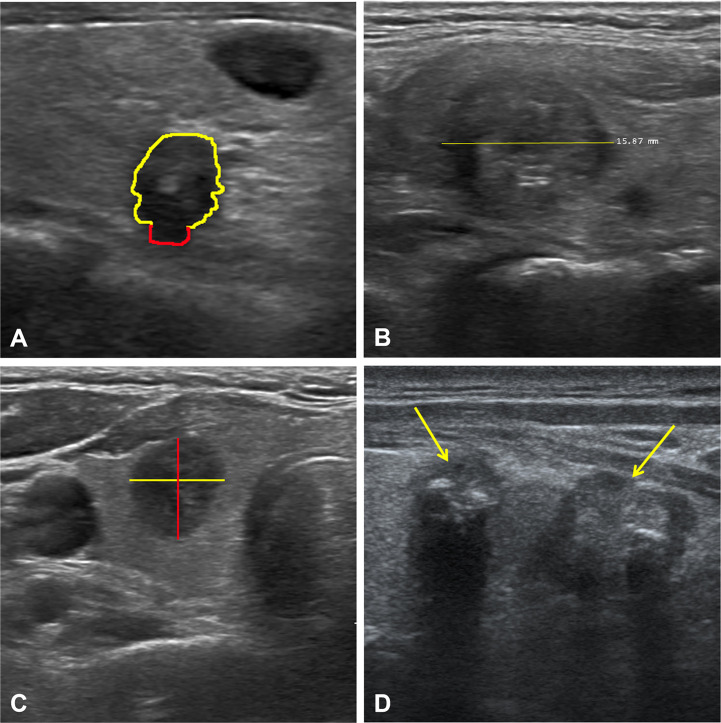
Ultrasound images of four representative cases. Case 1 **(A)**: The nodule with extrathyroidal extension (red line). Case 2 **(B)**: A solid nodule with a diameter of 1.6 cm. Case 3 **(C)**: A hypoechoic nodule with taller-than-wide. Case 4 **(D)**: Multifocality in the left lobe of the thyroid (yellow arrows). Together, all these features were the most essential factors in the diagnostic performance of the RF algorithm based on ultrasound features. RF, random forest.

The diagnostic performance of the RF algorithm was most dependent on the following five top-rank features, according to their mean decrease in Gini: ETE (27.597), age (17.275), T stage (15.058), shape (13.474), and multifocality (12.929) (**Figure 4**). Together, all these features were the most critical factors in the RF algorithm’s diagnostic performance based on ultrasound features (**Figure 5**).

## Discussion

We developed six machine learning models in the current study to differentiate CLNM and non-CLNM in PTC patients based on preoperative ultrasound. There were three significant findings. First, the six machine learning models could distinguish CLNM from non-CLNM based on preoperative ultrasound images to some extent. Second, after comparing the six machine learning models, RF had the best prediction performance. Third, the five most important factors affecting RF’s diagnostic performance were ETE, age, T stage, shape, and multifocality.

Presently, there were many studies (27–32) on the differential diagnosis of benign and malignant thyroid nodules using machine learning, but there were only a few studies applying machine learning models to predict lymph node metastasis in PTC patients. Some studies (19, 33) exploited the deep learning model to diagnose cervical lymph node metastasis in thyroid cancer patients with computed tomography (CT), and the highest AUC was up to 90.4%. However, there is still controversy about whether or not to routinely perform CT examinations for patients with thyroid cancer internationally due to the possible impact on subsequent radioactive iodine treatment (34). Lee et al. (35) used a deep learning-based computer-aided diagnosis system for localization and diagnosis of metastatic lymph nodes in thyroid cancer patients on ultrasound, and the accuracy was up to 83.0%. But they did not compare multiple machine learning models’ performance in distinguishing metastatic lymph nodes in patients with thyroid cancer.

By training six popular machine learning classifiers based on preoperative ultrasound images to identify which one would best differentiate between CLNM and non-CLNM, we found that the RF algorithm performed best. The RF classifier was more reliable for determining CLNM by comparing it with single ultrasound features. RF was a well-known machine learning algorithm for classification tasks and had an inherent resistance to overfitting, which was an ensemble learning method. It chose random data points from the data set to build multiple decision trees and improved the final prediction performance. We applied stratified 10-fold cross-validation in the current study, which randomly divided all the data into ten parts and then held out 10% of the testing data, repeated ten times.

ETE, age, T stage, shape, and multifocality were the five most important factors affecting the CLNM diagnostic performance of RF. In our study, ETE and multifocality were also associated with CLNM, indicating that the tumor was much more aggressive, which was consistent with previous studies (36, 37). The age of 55 was considered a watershed age in the TNM staging system of the 8th AJCC (26). Li et al. (11) reported that life expectancy was reduced in patients with thyroid cancer ≥45 years (cut-off value determined by the 7th AJCC). CLNM was closely related to the patient’s prognosis. In the current study, age ≥55 years was a significant independent risk predictor of CLNM, which was consistent with the literature (38). The diameter was recognized as an independent risk factor for CLNM in patients with PTC (10, 11, 36), and our study got the same result. It may be attributed to the more extensive the tumors, the more aggressive and proliferative. Taller-than-wide was another specific for distinguishing CLNM from non-CLNM in patients with PTC, which conveyed that malignant nodules grew across regular tissue planes, while benign nodules grew parallel to normal tissue planes (36, 39).

There were five limitations in this study. First, because it was a retrospective study, it might result in a potential selection bias. Thus, a multi-center much larger sample size prospective clinical research was required in the future. Second, the images’ quality had some variability because four radiologists re-evaluated the ultrasound images; however, all radiologists had rich experience in ultrasound diagnosis of thyroid cancer and followed TI-RADS (25) for image evaluation. Third, only patients diagnosed with PTC for the first time were enrolled; however, the sonographic features of thyroid bed recurrences might be significantly different, which is one of our future research directions. Fourth, the ultrasound reporting was not standardized prospectively, i.e., ideally, each examination should follow the same procedure and record the same dataset. Fifth, external validation was needed to conduct to verify the accuracy and reliability of the machine learning models in the future.

In conclusion, our study revealed that the machine learning-assisted ultrasound examination yielded a satisfactory performance in diagnosing CLNM in patients with PTC. Among these six machine learning models, RF had the best prediction performance. To our knowledge, this was the first study involving multiple machine learning classifiers specific for CLNM based on ultrasound. We expected that a machine learning model with better performance could help distinguish metastatic lymph nodes on ultrasound and provide a simple method for clinical, surgical decision-making in the future.

## Data Availability Statement

The raw data supporting the conclusions of this article will be made available by the authors, without undue reservation.

## Ethics Statement

The studies involving human participants were reviewed and approved by the Binzhou Medical University Hospital. Written informed consent for participation was not required for this study in accordance with the national legislation and the institutional requirements.

## Author Contributions

YZ, YS, JL, and FS contributed to the conception and design of the study and wrote the draft of the manuscript. GC and ML organized the database. YZ performed the statistical analysis. All authors contributed to the article and approved the submitted version.

## Conflict of Interest

The authors declare that the research was conducted in the absence of any commercial or financial relationships that could be construed as a potential conflict of interest.
