# Supplementary material for: A Comparative Analysis of Six Machine Learning Models Based on Ultrasound to Distinguish the Possibility of Central Cervical Lymph Node Metastasis in Patients With Papillary Thyroid Carcinoma
Source: Front Oncol. 2021 Jun 25;11:656127. doi: 10.3389/fonc.2021.656127 (PMC8270759; doi:10.3389/fonc.2021.656127)
Supplement: Supplementary file 1 [file DataSheet_1.docx]

**Appendices**

**Appendix: Ultrasound examination and image acquisition**

Phillips iU22, LOGIQ E9, SuperSonic Images AxiPlorer, Mylab Tweice, RS80A, and Mindray DC-8 color Doppler ultrasonic diagnostic apparatus were used to perform the ultrasound examination, equipping with L4-15 linear array probe, and the frequency was 4-15Hz. All patients were scanned in the supine position with shoulder pillows and their necks fully exposed; along with longitudinal and transverse scans, routine examination of the bilateral thyroid lobe, isthmus, and bilateral cervical lymph nodes was performed. Record the images in the picture archiving and communication systems (PACS).

**Table A.1: A brief description of six machine learning models**

| Model | Description |
| --- | --- |
| C5.0 | Decision Tree was a primary classification and regression method. C5.0 was one of the classic decision tree model algorithms, which can generate multi-branch decision trees, and the target variables were categorical. |
| LRA | A statistical method was used to analyze a dataset in which one or more independent variables determine the outcome. The outcome was measured by a dichotomous variable, in which there were only two possible outcomes. |
| SVM | It is a machine learning approach based on the structural risk minimization principle of statistics learning. It projected data into a multidimensional space and classified it with hyperplanes. |
| BN | An uncertainty processing model that simulated the causal relationship in the human reasoning process and its network topology was a directed acyclic graph. |
| ANN | It is a computational model based on the structure and function of biological neural networks. The main advantage of ANN was the ability to approximate any nonlinear mathematical function. |
| RF | An ensemble machine learning method for classification and regression, which operated by constructing a large number of decision trees and outputting classes as a single tree (classification) or average prediction (regression) model |

C5, decision tree C5.0 algorithm; LRA, logistic regression analysis; SVM, support vector machine; BN, Bayesian network; ANN, artificial neural network; RF, random forest.

**Figure Legends for the appendices**

Figure A.1: The mixed ROC curves of the six machine learning models in the training cohort. ROC, receiver operating characteristic; AUC, the area under the curve; C5.0, decision tree C5.0 algorithm; LRA, logistic regression analysis; SVM, support vector machine; BN, Bayesian network; ANN, artificial neural network; RF, random forest.

Figure A.2: The mixed ROC curves of the six machine learning models in the validation cohort. ROC, receiver operating characteristic; AUC, the area under the curve; C5.0, decision tree C5.0 algorithm; LRA, logistic regression analysis; SVM, support vector machine; BN, Bayesian network; ANN, artificial neural network; RF, random forest.

Figure A.3: The confusion matrices of the RF model in the training, validation, and test cohorts. RF, random forest.
