# Supplementary figures and images for: A Comparative Analysis of Six Machine Learning Models Based on Ultrasound to Distinguish the Possibility of Central Cervical Lymph Node Metastasis in Patients With Papillary Thyroid Carcinoma
Source: Front Oncol. 2021 Jun 25;11:656127. doi: 10.3389/fonc.2021.656127 (PMC8270759; doi:10.3389/fonc.2021.656127)

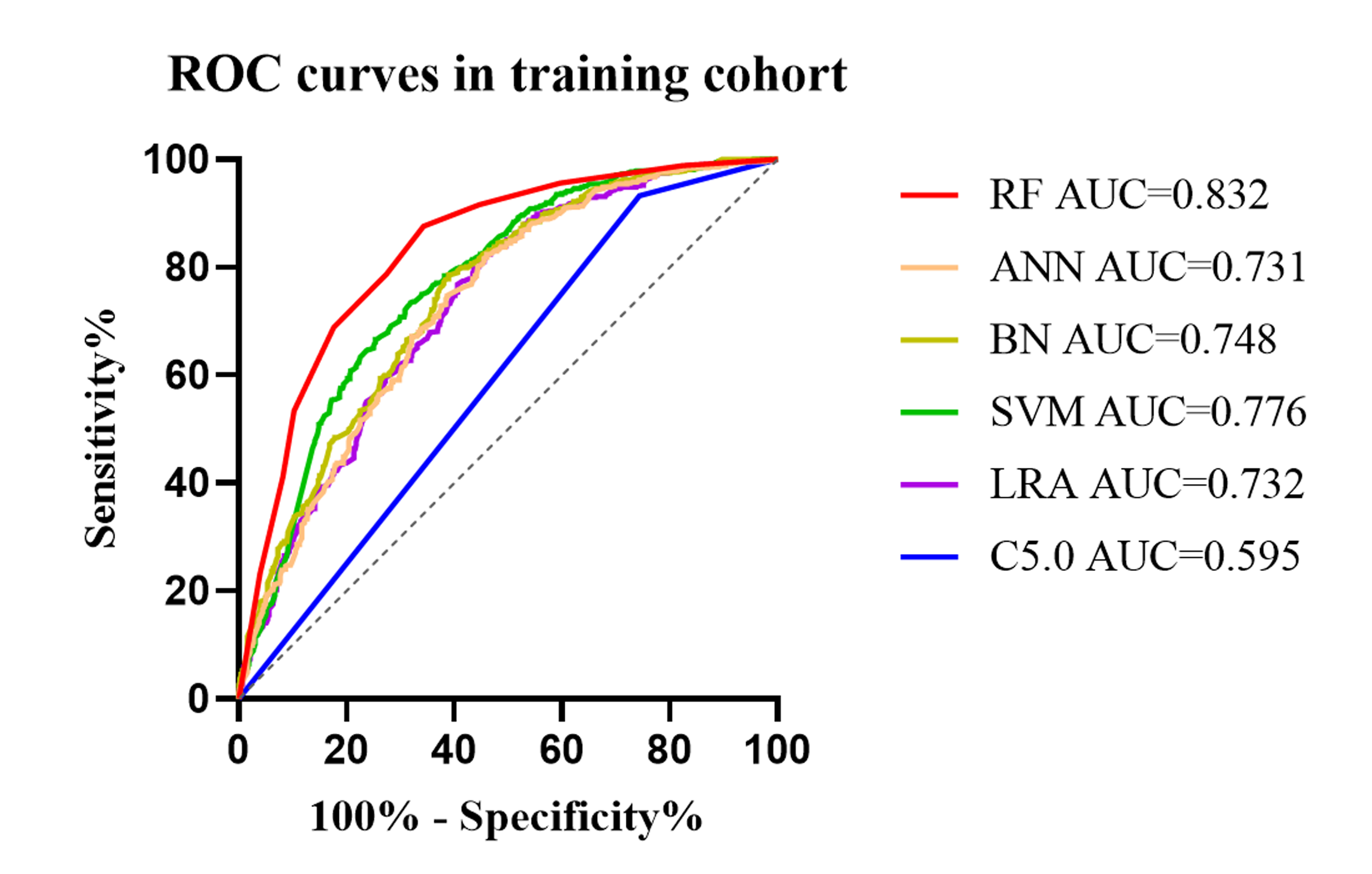

Supplement: Supplementary file 2 [file Image_1.tif]

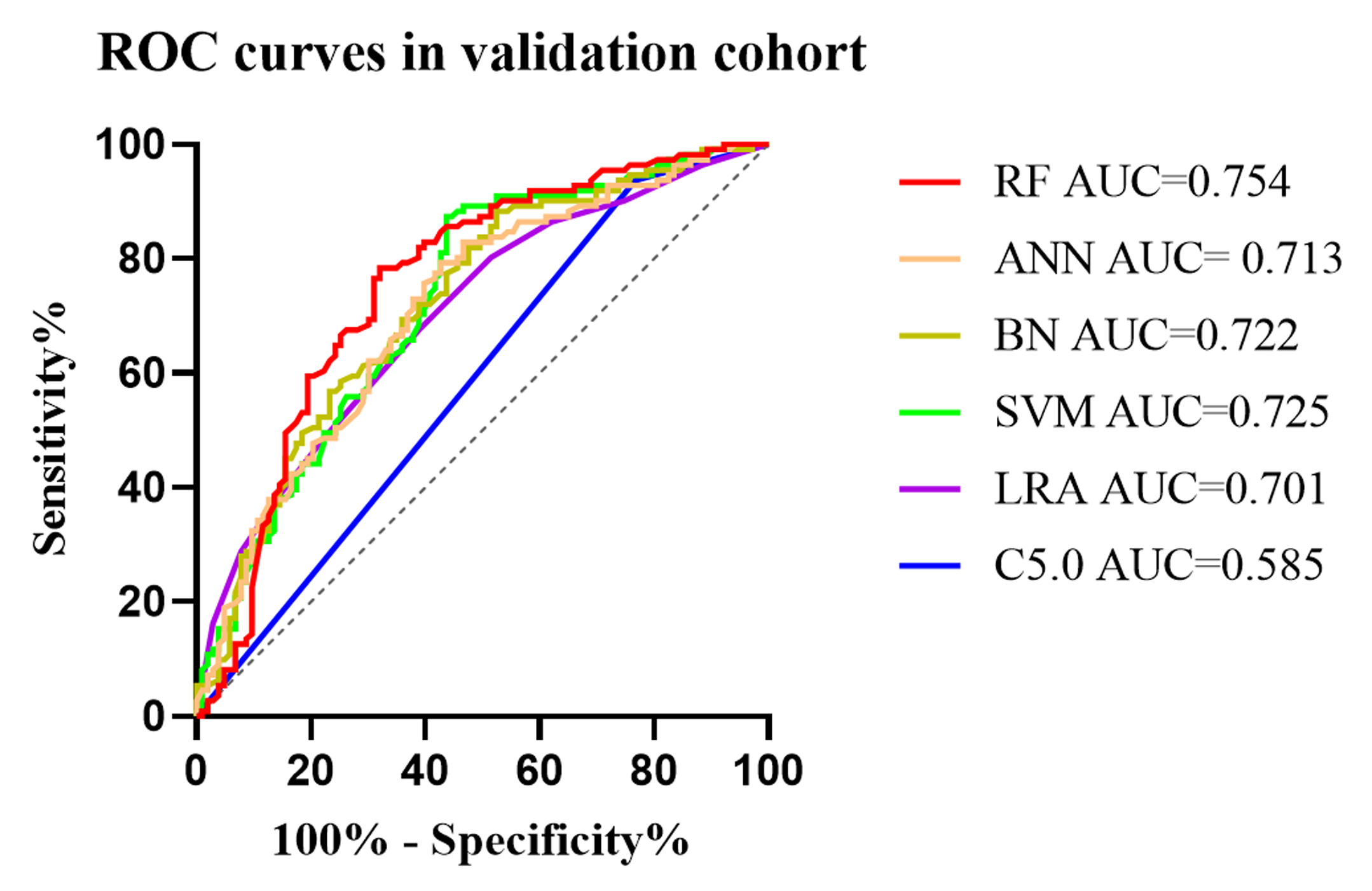

Supplement: Supplementary file 3 [file Image_2.tif]

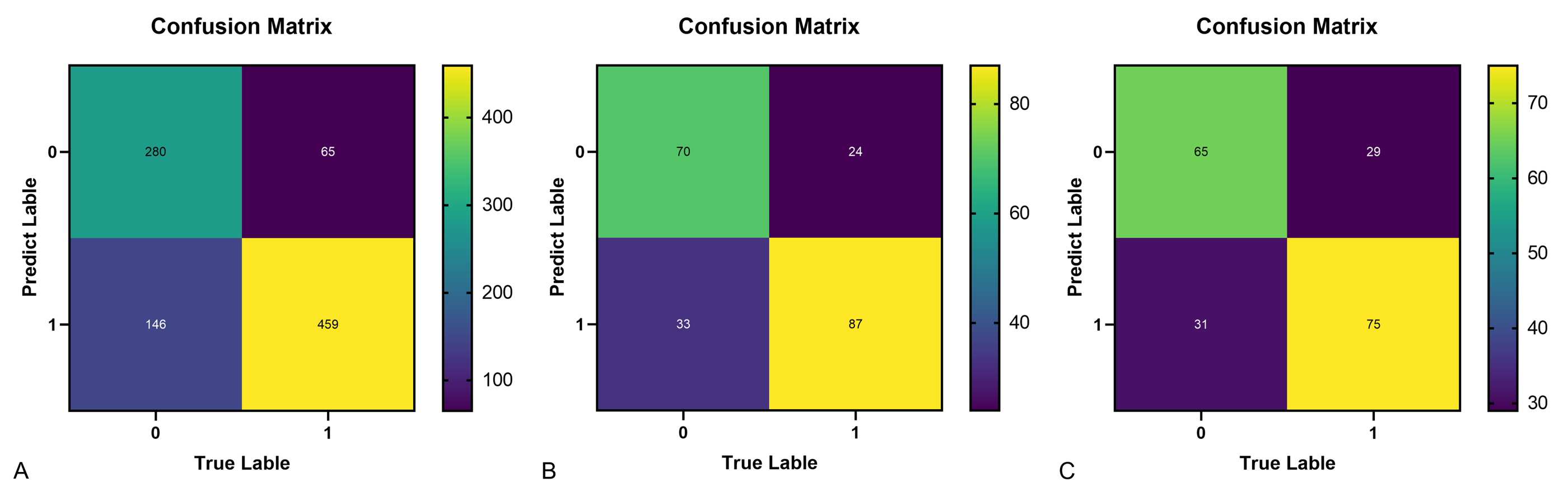

Supplement: Supplementary file 4 [file Image_3.tif]
